# Empirical investigations into Kruskal-Wallis power studies utilizing Bernstein fits, simulations and medical study datasets

**DOI:** 10.1038/s41598-023-29308-2

**Published:** 2023-02-09

**Authors:** Jeremy S. C. Clark, Piotr Kulig, Konrad Podsiadło, Kamila Rydzewska, Krzysztof Arabski, Monika Białecka, Krzysztof Safranow, Andrzej Ciechanowicz

**Affiliations:** 1 Department of Clinical and Molecular Biochemistry, Pomeranian Medical University, Ul. Powstancow Wlkp. 72, 70-111 Szczecin, Poland; 2 Department of Pharmacokinetics and Therapeutic Drug Monitoring, Pomeranian Medical University, Szczecin, Poland; 3 Department of Biochemistry and Medical Chemistry, Pomeranian Medical University, Szczecin, Poland

**Keywords:** Biological techniques, Medical research

## Abstract

Bernstein fits implemented into R allow another route for Kruskal-Wallis power-study tool development. Monte-Carlo Kruskal-Wallis power studies were compared with measured power, a Monte-Carlo ANOVA equivalent and with an analytical method, with or without normalization, using four simulated runs, each with 60–100 populations (each population with N = 30,000 from a set of Pearson-type ranges): random selection gave 6300 samples analyzed for predictive power. Three medical-study datasets (Dialysis/systolic blood pressure; Diabetes/sleep-hours; Marital-status/high-density-lipoprotein cholesterol) were also analyzed. In three from four simulated runs (run_one, run_one_relaxed, and run_three) with Pearson types pooled, Monte-Carlo Kruskal-Wallis gave predicted sample sizes significantly slightly lower than measured but more accurate than with ANOVA methods; the latter gave high sample-size predictions. Populations (run_one_relaxed) with ANOVA assumptions invalid gave Kruskal-Wallis predictions similar to those measured. In two from three medical studies, Kruskal-Wallis predictions (Dialysis: similar predictions; Marital: higher than measured) were more accurate than ANOVA (both higher than measured) but in one (Diabetes) the reverse was found (Kruskal-Wallis: lower; Monte-Carlo ANOVA: similar to measured). These preliminary studies appear to show that Monte-Carlo Kruskal-Wallis power studies, based on Bernstein fits, might perform better than ANOVA equivalents in many settings (and provide reasonable results when ANOVA cannot be used); and both Monte-Carlo methods appeared to be considerably more accurate than the analytical version analyzed.

## Introduction

There are few freely available methods for power analysis using Kruskal-Wallis tests. Some authors have suggested estimation of power and sample size using permutation methods, i.e. generating random datasets from a prespecified distribution with given input parameters. Using permutation, Hecke^1^ compared simulated populations with normal, lognormal and chi-squared distributions and found that Kruskal-Wallis tests compared favorably with analysis of variance (ANOVA) and it has long been known that, if medians/ranks are to be tested, the Kruskal-Wallis test is competitive to the *F*-test^2^.

Many alternative methods have been proposed. Mahoney and Magel^3^ tested four underlying continuous distributions that possessed various location configurations, to estimate power for Kruskal-Wallis analyses utilizing bootstrapping techniques to produce power estimates based on empirical cumulative distribution functions. The preferred configuration, the extended average X and Y method, was reliable for three groups and for more groups power was believed to be overestimated.

For continuous data, Fan et al.^4^ did not pursue the average X and Y method, but proposed and tested alternative methods: the AdjGeneric method and the shift g method—both thought to provide better estimates of power than F tests. The shift g method of Fan et al.^4^ has, as far as we know, not been implemented in R. In 2012, Fan and Zhang^5^ extended their work to encompass ordinal data and concluded that their method performed better than various Monte-Carlo methods if the underlying group distributions were not known (sample sizes needed to be larger than 100).

Several aforementioned algorithms have not so far, to our knowledge, been implemented in the R statistical platform packages and there appears to be no user-friendly tool freely available for the calculation of power and predicted sample sizes which utilizes the Kruskal-Wallis test. There is also the possibility that some Monte-Carlo methods, where predicted distributions or fits are created and then sampled at increasing sample size to determine power, might be more accurate with real-world data than some analytical methods.

From an initial pilot study with mean differences between three study groups, a power study can use characteristics of these groups to create simulated populations or fits, randomly sample these and estimate sample size for a statistically significant difference by Kruskal-Wallis tests. The algorithm proposed and implemented utilizes Bernstein fits of pilot study data. For this we rely heavily on the work performed by Hothorn et al., including the simulation testing of Bernstein fits^6,7^, who state that "models for the unconditional or conditional distribution function of any univariate response variable can be set up and estimated in the same theoretical and computational framework simply by choosing an appropriate transformation function and parameterization thereof"^6^. Bernstein fits, implemented as "most likely transformations"^7^, are likely to be competitive in terms of fitting non-normally-distributed real-world data.

In the present study for the purposes of simulation, up to 180 simulated populations from different Pearson-type distributions were analyzed: with different skewness/kurtosis combinations, plus populations with one group with normal moments, plus some analyses where ANOVA was performed on transformed data. For assessments with real data, three reasonably sized medical study datasets were analyzed: blood pressure measurements from a retrospective review of dialysis center medical records; numbers of hours of sleep versus diabetes classes from a multi-center epidemiologic study; and HDL-C measurements versus marital status from a multi-center cohort study.

For power comparison the created simulated datasets, plus the three medical datasets, provided "populations" from which initial samples of small size were drawn for "prediction" of sample size needed, and the populations were sampled at increasing sample size to give "measured" actual sample size at required power. Tools for Monte-Carlo Kruskal-Wallis power studies utilising Bernstein fits are provided for immediate use (Supplementary Files S7, S8).

The main aim of this paper was to perform comparisons between a Monte-Carlo ANOVA power study, an analytical ANOVA power study, a Monte-Carlo Kruskal-Wallis power study based on Bernstein fits, and measured power, using simulated datasets of various Pearson types, plus three medical study datasets.

## Methods

### Simulation studies

Relevant file at https://github.com/Abiologist/Power.git: S1B_Simulation_settings.R (see Supplementary Materials). Populations for each simulation run were produced with usually 10 000 values for each group and empirically-defined Pearson types (*PearsonDS::rpearson*^8^) within tolerance (rather than being drawn from infinite type populations; see Table 1 and Acknowledgements; note that processing was not possible for all types).

**Table 1:** A. Definitions of types of populations (POPtype) analyzed, including Pearson types ("POPtype" columns are also found in the skewdf dataframe and in mydfsample samples files). Populations with POPtype suffix including "3", e.g. B3a, had instructions for normalisation. B**.** Run absolute effect sizes and variances for all population groups together (Gall) and groups G1, G2 and G3. (run_onerelaxed created similarly to run_one but without reference to ANOVA assumptions.) Populations with POPtype "N" suffix had group G3 with normal moments.

A. POPtype	Skewness	Kurtosis	Pearson type	runs including this POPtype
Aa	0	1.1	II	not analyzed
Ba or B3a	0	3	0	all sim. runs, run_Dial, run_DIAB
Bb or B3b	0	5	VII	all sim. runs
C1a or C3a	1	5	V	run_one, run_two, run_Mar
C1b or C3b	1	3	I	not analyzed
D1a or D3a	0	10	VII	run_one
D1b or D3b	1	10	IV	run_one, run_two
E1a or E3a	2.14	10	III	run_one
E1b or E3b	2.14	15	IV	not analyzed
F	3	15	I	not analyzed

B. Run	Population means (medians)*: G1; G2; G3; Gall	Population variances (MADs)*: G1; G2; G3; Gall	Population types (POPtypes**, see Table 1A) included in run	
run_one	100; 100-0.5; 100-1; n.d	5, 5, 5, n.d	Ba Bb B3a B3b BaN BbN C1a C1aN C3a D1a D1aN D1b D1bN D3a D3b E1a E1aN E3a	
run_one - relaxed	100; 100-0.5; 100-1; n.d	5, 5, 5, n.d	Ba Bb B3a B3b BaN BbN C1a C1aN C3a D1a D1aN D1b D1bN D3a D3b	
run_two	100; 100–0.3; 100–0.55; n.d	5, 5, 5, n.d	Ba Bb B3a B3b BaN BbN C1a C1aN C3a D1b D1bN D3b	
run_three	100; 100-1; 100-2; n.d	20, 20, 20, n.d	Ba Bb B3a B3b BaN BbN	
run_Dial	135 (135); 132 (130); 127 (130); 132 (130)	452 (15); 341 (10); 434 (10); 427 (10)	Ba	
run_DIAB	8.02 (8); 7.82 (7.86); 7.92 (8); 7.92 (8)	1.99 (0.929); 1.96 (0.857); 2.31 (1); 2.05 (1)	Ba	
run_Mar	50.0 (47); 55.4 (53); 52.3 (50); 50.7 (48)	247 (10); 231 (10); 249 (10); 248 (10)	C1a	

*MAD* Median absolute deviation (R *mad(, constant* = *1)*); n.d. = not determined; * For simulation runs, means and variances were set (these were not determined for all groups together and medians/MADs were not determined); for medical study runs (run_Dial, run_DIAB and run_Mar): means, medians, variances and MADs were measured. ** POPtypes were set for simulation runs or approximated for medical study runs. For run definitions see text. sim. = simulation.

For each run a particular population variance was applied (Table 1) and mean differences were adjusted to give samples within reasonable time. Instructions for type production were saved in a dataframe "skewdf" and later passed to the list of dataframes "mydf" which also contained population data. In particular, note the skewdf column "ExpectNormal" had possible values "Norm", "Not-norm" or "KruskalOnly", according to whether it was reasonably possible (within time constraints) to produce samples (or populations) with requirements for ANOVA ("Norm"), for ANOVA with transformed variable ("Not-norm"); or only with Kruskal-Wallis requirements ("KruskalOnly").

Each population had all groups with the same Pearson type (defined in dataframe "skewdf1" and listed in "mydf1"), or with one group (G3) with normal moments (in skewdf2 and mydf2). Population copies of skewdf1/mydf1 were created with instructions for later normalization (in skewdf3 and mydf3). Dataframes skewdf1, skewdf2 and skewdf3 were combined to give skewdf and dataframe lists mydf1, mydf2 and mydf3 were combined to give mydf.

Selection criteria for each population were: (a) a statistically significant difference (with alpha defined for the populations; code: "alphaPOP") by Kruskal-Wallis test for differences among population groups; (b) Kruskal-Wallis power (with alpha specific for samples; "alpha") of 100 samples with size "initialsizeN" not found to be above specified power (80% or 90%; code: "power"); and (c) for certain population types where analyses using ANOVA or ANOVA of transformed data (skewdf column "ExpectNormal" with values "Norm" or "Not-norm") were expected to be used, ANOVA power (with "alpha") of 100 samples with size "initialsizeN" not found to be above specified power ("power").

### Medical study datasets

*Study (1). Dialysis study (code: run_Dial)*

Relevant files at https://github.com/Abiologist/Power.git: S1A_Dialysis_settings.R; S9A_Dialysis_data.csv. Systolic blood pressure measurements (mmHg; N = 15 062) with three groups of dialysis centers. Significant differences were previously found among 10 dialysis centers in Poland ^9^; here those with highest, medium and lowest blood pressure measurements were deliberately grouped together to give three groups with a statistically significantly difference among groups.

*Study (2). Diabetes study (code: run_DIAB)*

Relevant files: S1C_DIABETES_settings.R; S9C_DiabetesData.csv. Self-reported number of hours of sleep (h/day; N = 13 362; parameter "SLPDUR") with three American Diabetes Association diabetes classes (parameter "diabetes2"): 1, no diabetes; 2, pre-diabetes; and 3, treated diabetes; from the Hispanic Community Health Study (https://sleepdata.org/datasets, NHLBI National Sleep Science Resource^10^).

*Study (3). Marital study (code: run_Mar)*

Relevant files: S1D_Marital_settings.R; S9D_MaritalData.csv. Serum high-density-lipoprotein-cholesterol levels (HDL-C; mg/dL; N = 5079, parameter "hdl") and marital status (parameter "mstat": 1, married; 2, widowed; 3, divorced/separated (the small categories 4, never married and 8, unknown/refused, were removed); from the Sleep Heart Health Study (https://sleepdata.org/datasets, NHLBI National Sleep Science Resource^11^).

### Coding organisation

The master file S2A_SAMPLE_MASTER.R was run in R (R 4.0.3 GUI 1.73) to stitch a settings file (see relevant file in Study above) with the coding section 2 file (S2B_Sample_Coding_ section_2.R) to produce a sample coding file (SampleCoding) which was run on a single processor (Macbook Air, macOS Catalina 10.15.7, processor 1.8 GHz, memory 5 GB).

In this article "TRIAL" is used to refer to predictive power studies from samples with sample size "initialsizeN" whereas "MEASURE" is used to refer to measurements of power at increasing sample sizes starting at "initialsizeN".

Batches of SCRIPT files were produced in which each SCRIPT file was designed to analyze a number of samples; all results from one batch to be collated later. To produce these, another master file (S2C1_POWER_ MASTER.R) stitched the S3_Load_Coding.R file to one of three power coding files: for analysis parts 1 or 2 (depending on the settings): S4_Power_Coding_ parts1and2_Krus.R; for part 3: S4_Power_ Coding_part3_ANOVA.R or for part 4: S4_Power_Coding_ part4_MEASURE.R. This created SCRIPT files for one of four analyses: part 1: Kruskal Wallis TRIAL division algorithm (code: KRTDIV); part 2: Kruskal Wallis TRIAL duplicate algorithm (code: KRTDUP); part 3: ANOVA TRIAL (code: ANT) or part 4: Kruskal-Wallis or ANOVA MEASURE (codes: KRM or ANM). Occasionally (following timeout or memory failure) it was necessary to run another master file (S2C2_POWER_MASTER_SUBSET.R) to produce SCRIPT files each designed to analyze a smaller numbers of samples.

An Eagle Cluster (CPU E5-2697 v3 @ 2.60 GHz haswell; Ethernet 100/1000; InfiniBand FDR; Lustre storage; Operating System: CentOS Linux release 7.6.1810) at the Poznan Supercomputing and Networking Center (Poznan, Poland; https://hpc.man.poznan.pl) was accessed by including R cluster functions in the script: *parallel::makeCluster*^12^ and *doParallel::registerDoParallel*^13^. The SCRIPT file main loop was implemented as a function containing R *foreach::foreach*^14^ with set seeds distributed using R [doRNG]^15^ functions: *registerDoRNG*, *.options.RNG* and *%dorng%*.

Files with simulated samples or data for medical studies, R scripts, and slurm coding (e.g. S5_aaSLURM _sim_run_two_part2.sl) were passed (using Forklift 3.4.4; https://www.torrentmac.net/forklift-3-4-4) to the cluster and run for a maximum of 4 h.

Result files (file part-name: "datastore") were collated using a collation file (S6_Collation_Coding.R). Graphics were produced using R ggplot2^16^ (graphical coding found in file S10_GRAPH_NON _PARAMETRIC.R) plus Designworks version 3.5 (Greenstreet Software, Huntingdon, UK). All references for R libraries are given at the end of file S1A.

### Sample-selection coding

Relevant file: S2B_Sample_Coding_section_2.R. For all studies from each population, 10 or 30 samples were created, each without a statistically significant difference among groups by Kruskal-Wallis test (plus by ANOVA except for populations designated as "relaxed" or with a skewdf value of "KruskalOnly"). The sample sizes (of all groups together; termed "initialsizeN") of these samples were chosen (arbitrarily) at around 1/3 sample size of that for preliminary measured power of 90%. Samples from populations with a skewdf value of "Norm" were selected to be normally distributed (using R [stats] *shapiro.test*); with a skewdf value of "Not-norm" samples were selected so that transformed samples (transformed using *jtrans::jtrans* or *lamW::lambertW0*) were normally distributed; with a skewdf value of "KruskalOnly" no requirement for normality was applied. Skewness, kurtosis, and other settings for all runs are found in Table 1.

### Predicted Sample Sizes (codes: TRIAL, KRTDUP, KRTDIV, ANT)

For Kruskal-Wallis predicted power, Bernstein fits were found for samples using R [mlt]^6^ functions: *numeric_var*, *as.basis*, *ctm* and *mlt*. Fits were found separately for each of the three groups and in order to do this (with *mlt* version 0.2) for each group *as.basis* was fed a dataframe with a two-leveled factor in one column and the quantitative data in the second column. Two alternative algorithms were therefore implemented: either the group data was duplicated (KRTDUP), the duplicate relabelled with a different factor name and appended row-wise; or the group data were randomly divided (KRTDIV) into two near-equal parts and one part was relabelled and appended row-wise to the other.

The models used were:

numvar <—*numeric_var*("Group", support = c(min(df$Group), max(df$Group)))

basis <—*as.basis*(~ Factor - 1, data)

myctm <—*ctm*(response = Bernstein_basis(numvar, order = 4, ui = "increasing"), interacting = basis, data)

*mlt*(myctm, data, trace = TRUE, maxit = 10000)

With the latest version of R [mlt] (version 1.3–2) the last line must be changed as follows (this is implemented in the Kruskal tool (S8_KRUSKAL_TOOL.R) but other files give the original coding used to obtain the results):

*mlt*(myctm, data, optim = mltoptim(trace = TRUE, spg = list(maxit = 10000)))

Simulated samples were created from the fits (using *mlt::simulate*) at increasing sample sizes (starting from initial sample size "initialsizeN + 1") until required power.

For Monte Carlo ANOVA (with White adjustment) predicted power ("ANT"), normal distributions were fitted and sampled, again at increasing sample sizes. Effect sizes used Vargha and Delaney's A statistic.

### Actual Power (codes: MEASURE, ANM or KRM)

Actual sample sizes needed to obtain a particular power (by Kruskal-Wallis tests, KRM or ANOVA, ANM) were estimated by analyzing random samples, sampled from a population at increasing sample size, starting with initial sample size initialsizeN + 1. As with the Trials the samples drawn had the same (rounded) group proportions as in the population.

### Analytical

Analytical ANOVA power was found using R *stats::power.anova.test* (coding in S4_Power_Coding_ part4_ MEASURE.R).

#### Missing data

Missing data arose if sample power did not exceed required power, if an analytical model could not be formed or, for ANOVA measurements, if samples from particular population types were deliberately not analyzed (with these types, ANOVA assumptions would almost certainly be broken, analyses would take considerable time, and/or the results would give far from useful predictions).

### Statistics

All tests were two-tailed. Alpha for the Medical studies was set at *p* = 0.05. Differences between ANOVA and Kruskal-Wallis "trial" or "measured" power were tested for statistical significance using Brown-Mood median tests. Non-parametric aligned rank transform was also used to compare parameters (TRIALs, MEASURE, Analytical) and results are given in S10_GRAPH_NON_PARAMETRIC.R or S12_Medians_Moods.R. (Note that parametric comparisons were also attempted: Poisson, negative binomial and zero-inflated models, but these failed mostly due to excessive overdispersion).

## Results

**Figure 1:**
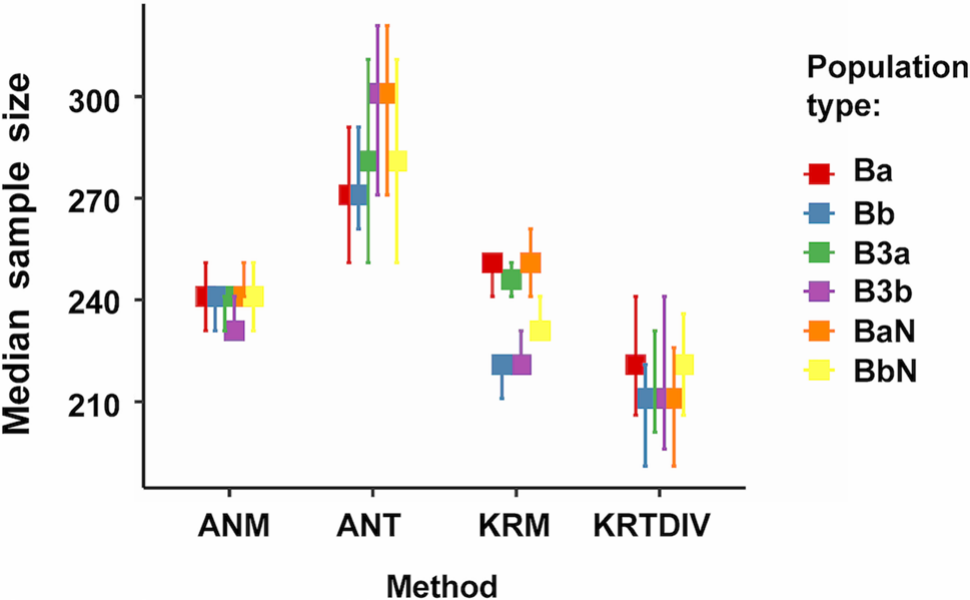
Simulation run_three: population group means: G1: 100; G2: 100-1; G3: 100-2; population variance: 20. Medians of measured (ANM: ANOVA, KRM: Kruskal-Wallis) and Monte-Carlo predicted (ANT: ANOVA, KRTDIV: Kruskal-Wallis) sample sizes for 80% power. Error bars: 95% confidence intervals. For run settings, algorithms and Population type definitions, see Tables 1 and 2.

The main results from simulations studies are shown in Table 2 and Figs. 1:4 (which can also be generated by running S10_GRAPH_NON_PARAMETRIC.R). The two internal algorithms (duplication, KRTDUP or division, KRTDIV) for Monte-Carlo Kruskal-Wallis prediction gave similar results for all analyses and only KRTDIV is shown in these Figures. Sample sizes from the three prediction methods: KRTDIV; Monte-Carlo ANOVA (ANT); and analytical ANOVA power ("Analytical"); were compared with those of measured power from ANOVA (ANM) and/or Kruskal-Wallis tests (KRM). The best methods were presumed to be those with median sample sizes most similar to those from KRM and ANM (ANM sample sizes gave very similar distributions to KRM but had more missing data; note that modes shown in Supplementary Figures can be deceptively different from the medians or means).

**Table 2:** Predicted and measured sample sizes for 80% power using Monte-Carlo Kruskal-Wallis tests or Monte Carlo or analytical ANOVA, using samples derived from simulated populations.

Simulation run (N)	Sample size estimate for 80% power (median ± MAD, n)					
	ANM (measured)	ANT (predicted)	KRM (measured)	KRTDIV (predicted)	KRTDUP (predicted)	Analytical AN (predicted)
run_one (N = 1800)	230^ad^ ± 30, 1193 230 ± 30, 1200	280^abc^ ± 100, 1193 280 ± 100, 1395	230^ce^ ± 30, 1193 220 ± 40, 1800	210^bde^ ± 80, 1193 200 ± 70, 1798	n.a 200 ± 70, 1797	n.a 360 ± 150, 1398
run_one relax (N = 1500)	230^fi^ ± 30, 1493 230 ± 30, 1500	270^fgh^ ± 100, 1493 270 ± 100, 1495	220^hj^ ± 30, 1493 220 ± 30, 1500	200^gij^ ± 70, 1493 200 ± 70, 1498	n.a 200 ± 70, 1498	n.a 340 ± 150, 1500
run_two (N = 1200)	640^kp^ ± 90, 788 640 ± 90, 800	580^kmn^ ± 240, 788 580 ± 240, 893	650^nr^ ± 90, 788 640 ± 100, 1200	460^mpr^ ± 180, 788 450 ± 190, 1185	n.a 450 ± 180, 1185	n.a 830 ± 400, 900
run_three (N = 1800)	240^sv^ ± 30, 1790 240 ± 30, 1800	280^stu^ ± 100, 1790 280 ± 110, 1794	240^uw^ ± 30, 1790 240 ± 30, 1800	220^tvw^ ± 70, 1790 210 ± 70, 1794	n.a 220 + 80, 1794	n.a 360 ± 150, 1799

Results from all Pearson types pooled. *ANM* ANOVA, measured; *ANT* Monte-Carlo ANOVA trial, predicted; *KRM* Kruskal–Wallis, measured; *KRTDIV* Monte-Carlo Kruskal-Wallis trial, predicted (division algorithm); *KRTDUP* Monte-Carlo Kruskal-Wallis trial, predicted (duplication algorithm); *Analytical AN* Analytical ANOVA. See Table 1 for descriptions of simulation runs.

N = original number of samples; *The methods for ANT, ANM, KRTDIV, KRTDUP, KRM and Analytical ANOVA are defined in the text. Medians and MAD (= median absolute deviation around the median; R *mad(constant* = *1*) to two significant figures: for each run the first rows of numbers are for statistical comparisons between ANT, ANM, KRTDIV or KRM (samples were removed if any of these parameters had missing values; n = number of samples remaining), second rows include values from all samples, regardless of whether one parameter gave a missing value (n = number of non-missing values). Bonferroni-adjusted Brown-Mood median test comparisons with same letter significantly different at p < 0.05, other comparisons not significantly different: ^abfgprstuv^*p* < 1.32 × 10^−15^; ^a^Z = 8.23; ^b^Z = 8.88; ^c^Z = 7.78, *p* = 4.40 × 10^−14^; ^d^Z = 4.18, *p* = 0.000148; ^e^Z = − 3.4387, *p* = 0.00301; ^f^Z = 8.56; ^g^Z = 9.74; ^h^Z = 6.65, *p* = 1.72 × 10^−10^; ^i^Z = 7.77, *p* = 4.80 × 10^−14^; ^j^Z = − 6.04, *p* = 9.27 × 10^–09^; ^k^Z = − 3.63, *p* = 0.00173; ^m^Z = 5.44, *p* = 3.21 × 10^−07^; ^n^Z = − 4.03, *p* = 0.000335; ^p^Z = 12.0; ^r^Z = − 11.9; ^s^Z = 9.86; ^t^Z = 10.8; ^u^Z = 10.7; ^v^ Z = 9.08; ^w^Z = − 7.56, p = 2.36 × 10^−13^. n.a. = not applicable; run_one relax. = run_one relaxed.

From simulated run "run_three" (Figs. 1 and S13) with variance 20, group means ~ 98 to ~ 100, Pearson types 0 and VII (Table 2), KRTDIV pooled results (from all Pearson distributions) gave a median predicted sample size (all values in this section: median ± median absolute deviation) of 221 ± 70 which was 8% lower than that for measured power (KRM or ANM: 241 ± 30). This indicates that the Monte-Carlo Kruskal-Wallis power studies were fairly accurate but non-conservative. Monte-Carlo ANOVA (ANT) gave a pooled median sample size of 281 ± 110, i.e. 17% higher, and this was therefore less accurate overall than the Kruskal-Wallis method and conservative (note the lower median absolute deviation for KRTDIV than for ANOVA). The analytical method (see Fig. S13, Table 2) gave higher sample size values (357 ± 150). For these settings the amount of missing data was negligible (< 0.4% for all parameters).

**Figure 2:**
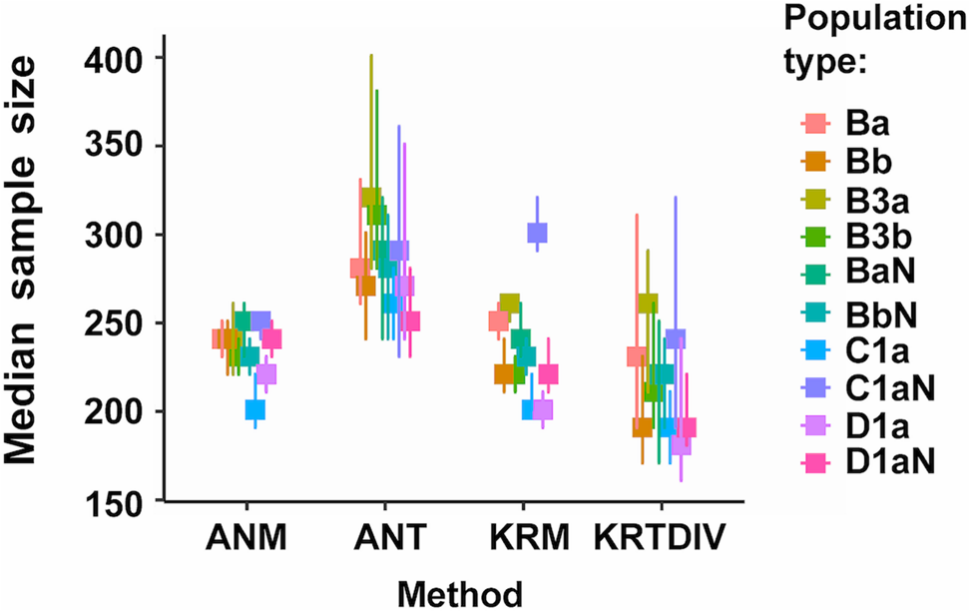
Simulation run_one: population group means: G1: 100; G2: 100-0.5; G3: 100-1; population variance: 5. Medians of measured (ANM: ANOVA, KRM: Kruskal-Wallis) and Monte-Carlo predicted (ANT: ANOVA, KRTDIV: Kruskal-Wallis) sample sizes for 80% power. Error bars: 95% confidence intervals. For run settings, algorithms and Population type definitions, see Tables 1 and 2.

From simulated run "run_one" (Figs. 2 and S17) with variance 5, group means ~ 99 to ~ 100 with many Pearson types (Table 2), KRTDIV pooled results (from all Pearson types) gave a median predicted sample size of 211 ± 80, 9% lower than that from measured power (KRM or ANM: 231 ± 30). Again, the Monte-Carlo Kruskal-Wallis method was fairly accurate but non-conservative. Monte-Carlo ANOVA (ANT) gave a pooled median sample size of 281 ± 100, i.e. 22% higher, and this was therefore less accurate overall than the Kruskal-Wallis method and conservative. The analytical method (see Fig. S17) gave higher sample size values (360 ± 153). For the settings of run_one the amount of missing data was not negligible: ANM or KRM: 0%; Analytical or ANT: 22%; KRTDUP or KRTDIV: < 0.2%. (Note that the median absolute deviation of sample sizes for KRTDIV was less than for the ANOVA methods).

**Figure 3:**
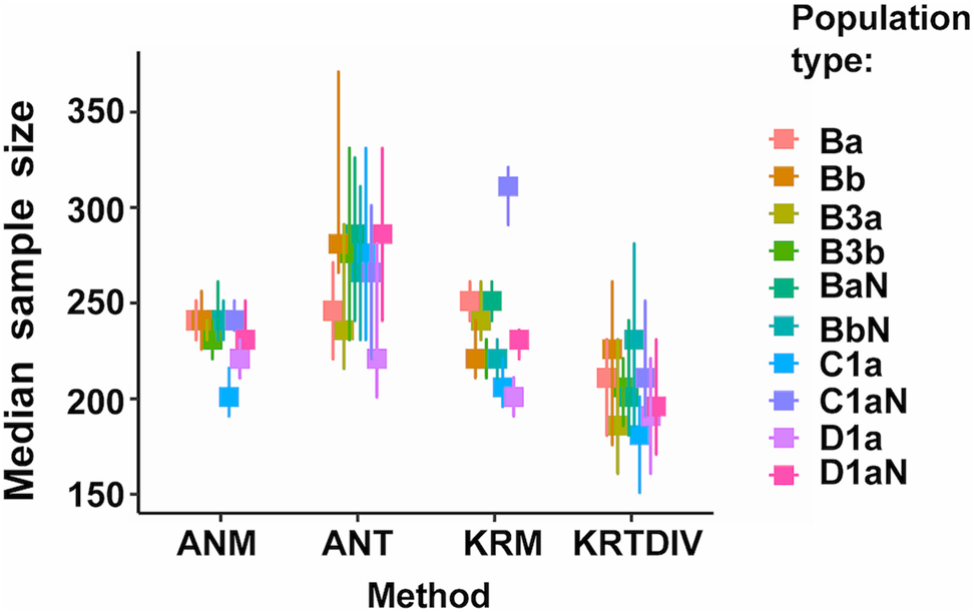
Simulation run_one relaxed: population group means: G1: 100; G2: 100-0.5; G3: 100-1; population variance: 5; relaxed = samples chosen regardless of whether they satisfied ANOVA assumptions. Medians of measured (ANM: ANOVA, KRM: Kruskal-Wallis) and Monte-Carlo predicted (ANT: ANOVA, KRTDIV: Kruskal-Wallis) sample sizes for 80% power. Error bars: 95% confidence intervals. For run settings, algorithms and Population type definitions, see Tables 1 and 2.

Simulated run "run_onerelaxed" was similar to run_one but with samples chosen with settings relaxed from ANOVA assumptions (Figs. 3 and S18), with the same range of means and variance as run_one and many Pearson types (Table 2), and might be regarded as providing the most realistic range of simulations. From run_onerelaxed, KRTDIV pooled results (from all Pearson types) gave a median predicted sample size of 201 ± 70, 11% lower than that for measured power (226 ± 30). Again, the Monte-Carlo Kruskal-Wallis method was fairly accurate but non-conservative. Monte-Carlo ANOVA (ANT) gave a pooled median sample size of 271 ± 105 i.e. 20% higher and was therefore less accurate overall than the Kruskal-Wallis method and conservative (note it could be argued it shouldn't be used at all for many of these populations !). The analytical method (see Fig. S18) gave higher sample size values (344 ± 155). For these settings the amount of missing data was negligible (< 0.4% for all parameters). (Note that the median absolute deviation of sample sizes for KRTDIV was less than for the ANOVA methods).

**Figure 4:**
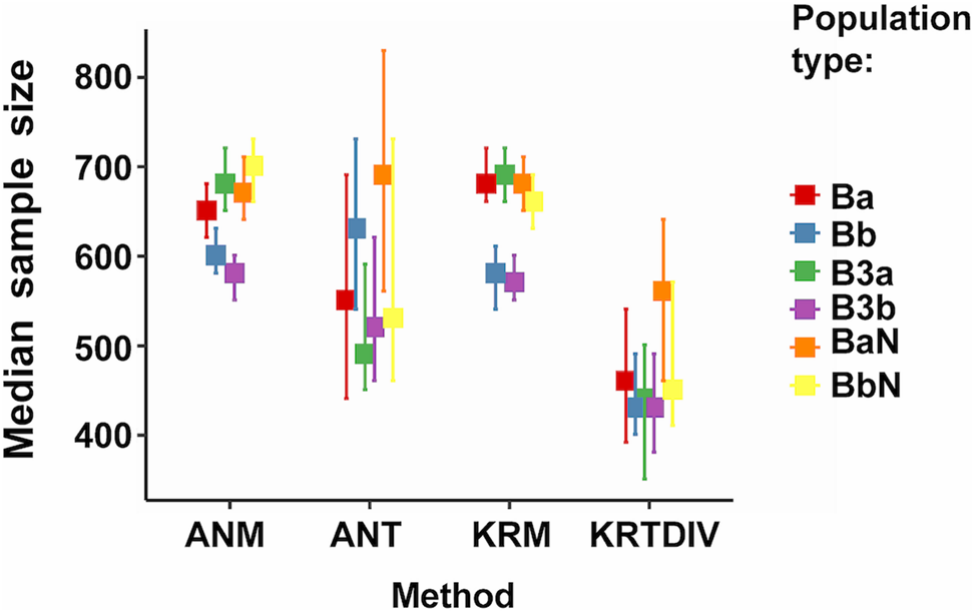
Simulation run_two: population group means: G1: 100; G2: 100-0.3; G3: 100-0.55; population variance: 5. Medians of measured (ANM: ANOVA, KRM: Kruskal-Wallis) and Monte-Carlo predicted (ANT: ANOVA, KRTDIV: Kruskal-Wallis) sample sizes for 80% power. Error bars: 95% confidence intervals. For run settings, algorithms and Population type definitions, see Tables 1 and 2.

From simulated run "run_two" (Figs. 4 and S19) with variance 5, group means ~ 99.45 to ~ 100 with many Pearson types (Table 2), KRTDIV pooled results (from all Pearson types) gave a median predicted sample size of 456 ± 180, 29% lower than that from measured power (646 ± 90). This time the Monte-Carlo Kruskal-Wallis method was less accurate and non-conservative. Monte-Carlo ANOVA (ANT) gave a pooled median sample size of 581 ± 240 i.e. 9% higher and was therefore more accurate than the Kruskal-Wallis method and conservative. The analytical method (see Fig. S19) gave higher sample size values (827 ± 389). For the settings in run_two the amount of missing data was not negligible: ANM or KRM: 0%; Analytical: 25%; ANT: 26%; KRTDUP or KRTDIV: < 2%. (Note that the median absolute deviation of sample sizes for KRTDIV was less than for the ANOVA methods).

If analyses were pooled for those settings with Pearson types which were difficult or impossible to process using ANOVA (see Table 3), the Kruskal-Wallis method appeared to do rather well for run_one, with predicted sample sizes very close to that measured by KRM, but less so for run_two, with predicted sample sizes around two thirds of those from measured power.

**Table 3:** Predicted (trial KRTDIV or KRTDUP algorithms) and measured (MEASURE) sample sizes for 80% power using Monte-Carlo Kruskal-Wallis tests, with samples derived from simulated populations for which ANOVA would be either difficult or impossible to process.

Simulation run, N	Sample size estimate for 80% power (median ± MAD, n)		
	KRM (measured)	KRTDIV (predicted)	KRTDUP (predicted)
run_one (N = 1800)	150 ± 40, 405 150 ± 40, 405	160 ± 50, 405 160 ± 50, 405	n.a 160 ± 50, 404
run_two (N = 1200)	670^a^ ± 130, 299 670 ± 130, 307	440^a^ ± 190, 299 440 ± 190, 299	n.a 450 ± 200, 299

Results from all Pearson types pooled from data for which ANOVA trial (ANT) predicted sample sizes were missing (either because of failure or not processed). *KRM* Kruskal-Wallis, measured; *KRTDIV* Monte-Carlo Kruskal-Wallis trial, predicted (division algorithm); *KRTDUP* Monte-Carlo Kruskal-Wallis trial, predicted (duplication algorithm); See Table 1 for descriptions of simulation runs.

N = original number of samples; Median and MAD = median absolute deviation around the median (R *mad(constant* = *1)*) to two significant figures: for each run the first rows of numbers are for statistical comparisons between KRTDIV and KRM (samples were removed if either of these had missing values; n = number of samples remaining), second rows include values from all samples, regardless of whether one parameter gave a missing value (n = number of non-missing values). run_one: no significant difference between KRTDIV and KRM medians by Brown-Mood median tests; run_two ^a^Z = − 7.36, *p* = 1.89 × 10^−13^. n.a. = not applicable.

The diabetes study had data with means ~ 7.8 to ~ 8, variance 2, Pearson type 0 and no power study method appeared to perform well (Fig. S14, Table 4; note that ANM either failed completely or was not processed). The median predicted sample size for KRTDIV (2300 ± 600) was significantly (26%) lower than that from KRM (3100 ± 300) whereas the median predicted sample size from ANT (3300 ± 1000) was not significantly different from that from KRM (see Table 4). Analytical ANOVA gave a wide range of predictions (5100 ± 3200).

**Table 4:** Predicted and measured Monte-Carlo sample sizes for 90% power using ANOVA or Kruskal-Wallis tests, using samples derived from Medical study result datasets.

Study and source	Type	Sample size estimate for 90% power (median ± MAD)					
		ANOVA,			Kruskal–Wallis***,		
		Sample sizes	Min. effect size**	No fails	Sample sizes	Min. effect size**	No fails
(1) Systolic blood pressure; 3 groups of dialysis centers^9^. N_D_ = 15,062; n = 197/200	Predicted,	770 ± 300^ab^	0.56 ± 0.021	3	570 ± 200^a^	0.56 ± 0.024	1
	Measured,	n.c	n.c	n.c	570 ± 100^b^	0.58 ± 0.011	0
(2) Number of hours of sleep; 3 diabetes classes^10^. N_I_ = 13,362; n = 79 / 100	Predicted,	3300 ± 1000^c^	0.52 ± 0.014	17	2300 ± 600^ cd^	0.52 ± 0.013	13
	Measured,	n.c	n.c	n.c	3100 ± 300^d^	0.53 ± 0.0031	0
(3) High-density-lipoprotein cholesterol; 3 marital status classes* ^11^. N_I_ = 5079; n = 185/200;	Predicted,	950 ± 260^efg^	0.50 ± 0.028	6	760 ± 160^fhj^	0.50 ± 0.031	4
	Measured,	460 ± 70^ehi^	0.52 ± 0.022	0	390 ± 40^gij^	0.52 ± 0.022	0

*ANOVA* Analysis of variance, *MAD* Median absolute deviation; Min. = minimum; No. fails = number of failures; Predicted: sample sizes with 90% power from predicted fits created from initial samples with (arbitrarily) approximately one third sample size. Measured: sample sizes with power measured at 90% from data randomly selected from datasets at increasing sample size. n = number of times prediction or measurement carried out. N_D_ = number of dialysis treatments; N_I_ = number of individuals. All results to two significant figures. Brown-Mood median test (R [coin] *median_test*) global values (2 df): (1) chi^2^ = 29.4, *p* = 4.07 × 10^−7^ (2) chi^2^ = 9.13, *p* = 0.0104 (3) chi^2^ = 506, *p* < 2.2 × 10^−16^; Bonferroni-adjusted Brown-Mood median test comparisons with same letter significantly different at *p* < 0.05, other comparisons not significantly different: ^a^Z = 3.46; *p* = 0.00171; ^b^Z = 4.65, *p* = 1.00 × 10^−5^; ^c^Z = 3.65, *p* = 0.000263; ^d^Z = 3.49, *p* = 0.00145; ^e^Z = 13.6, *p* < 1.32 × 10^−15^; ^f^Z = 4.26, *p* = 0.000124; ^g^Z = 18.2, *p* < 1.32 × 10^−15^; ^h^Z =− 12.8, *p* = < 1.32 × 10^−15^; ^i^Z = 7.38, p = 9.55 × 10^−13^; ^j^Z = 18.0, *p* < 1.32 × 10^−15^. *after Johnson transformation. **These are medians of minimum effect sizes detected or measured for each trial. ***Division algorithm i.e. KRTDIV. n.c. = not calculable e.g. sample size reached maximum.

The dialysis study had data with means ~ 127 to ~ 135, variance 427, Pearson type 0, and power studies appeared to perform well (Fig. S15; but note ANM either failed completely or was not processed; Table 4). The median sample size from KRTDIV (570 ± 200) was identical to that from KRM (570 ± 100), whereas the median sample size from ANT (770 ± 300) was significantly (35%) higher (Table 4), again showing that the Kruskal-Wallis method was more accurate. The analytical method gave high sample size values (670 ± 360).

With the Marital study (Fig. S16) sample production had to use a relaxed method (without considering ANOVA assumptions), and all samples were analyzed with Johnson- transformed ANOVA and Kruskal-Wallis methods. All prediction methods were significantly conservative i.e. with predicted sample sizes significantly higher than those from ANM (460 ± 70) or KRM (390 ± 40). KRTDIV (sample sizes 760 ± 160) performed slightly better than ANT (950 ± 260) and Analytical (2100 ± 970) performed considerably worse (Fig. S16; Table 4).

Result ("datastore") files contain considerable statistics (full parameter list in S11_Parameter_ Definitions.xlsx) including empirical moments, Vargha and Delaney's A effect sizes, and all p values and rank orders for final tests. Minimum detected effect sizes (predicted and measured) were similar for ANOVA and Kruskal-Wallis power studies.

## Discussion

Efficient study replication should involve power studies to estimate minimal sample sizes while minimising false negative frequencies.

The Kruskal-Wallis test is commonly applied to non-parametric studies with continuous variables and a derived power study theoretically has broad application. Bernstein fits have recently been implemented in R and therefore provide a basis by which Monte-Carlo Kruskal-Wallis power prediction can be formulated and, as well as having broader applicability, from the present study appear overall to be more accurate than ANOVA (both Monte-Carlo or analytical) methods even when conditions for ANOVA are fulfilled.

From the analyses in the present study using simulated or medical data, Monte-Carlo Kruskal-Wallis power prediction did not seem to be at any particular disadvantage when compared with Monte-Carlo ANOVA and both Monte-Carlo methods appeared considerably more accurate than the Analytical method used. (Note that with these methods ties in the data are allowed.) Under most circumstances the Monte-Carlo Kruskal-Wallis method was more accurate, even when normal distribution was demanded (run_one and run_three) and detected similar minimum effect sizes to the Monte-Carlo ANOVA method. It is possible, as suggested by run_two and the diabetes study, that in cases where the group means are very similar the Kruskal-Wallis method might be less accurate than Monte-Carlo ANOVA, although further studies are needed to show that this is indeed the case.

Overall, from these preliminary studies, it appears that Monte-Carlo Kruskal-Wallis power studies based on Bernstein fits might be able to be used in preference to ANOVA, and could probably be used if ANOVA failed or if assumptions were in doubt (see Table 3), over a much wider range of different samples. Additionally, both Monte-Carlo methods were more accurate overall than the analytical version assessed.

Further large-scale testing is needed to show the scope of conditions in which the accuracy of the Kruskal-Wallis method falls, and also its accuracy with, for example, truncated data, bimodal distributions or with further examples of unequal group sizes.

As in Fan et al.^4^ in the present study the simulations had equal group sizes, and it might well be the case that the Kruskal-Wallis method might perform better than ANOVA overall if sizes of individual groups were allowed to differ. It is unknown whether, as in Fan and Zhang^5^, the method will perform poorly with small group sizes.

The fact that the Kruskal-Wallis method compared favorably with ANOVA, both with samples from real datasets and from simulated populations with moderate skewness, leads us to suggest that, in the absence of a freely available alternative, the method can be used immediately, possibly in preference to ANOVA methods.

It will be intriguing to see the extent to which the Kruskal-Wallis method has advantage over ANOVA methods under a larger range of conditions and how the method compares with the (rather numerous) possible power-study alternatives (see Introduction and^1,3,5^). Further large-scale testing against these and other possible methods is advised, together with a larger range of simulated data.

We present these results and implemented code to provide simulation and empirical evidence for the utility of a freely available Monte-Carlo Kruskal-Wallis power method which utilizes Bernstein fits as developed by Hothorn et al.^17^. While the method can be used immediately, further testing is advised to determine the scope under which this method has advantage over ANOVA or where accuracy falls.

### Limitations

1. Analysis of further empirical datasets is needed in order to confirm the finding that the Monte-Carlo Kruskal method has some advantages over other methods.
2. Although the Kruskal-Wallis method can be used immediately, further large-scale simulation testing would allow delimitation of the conditions under which accuracy falls.
3. The method should also be compared with the AdjGeneric and the shift g method to determine comparative effectiveness.

## Conclusions

This preliminary study has shown that, with samples for which ANOVA can also be used, the Monte-Carlo Kruskal-Wallis method was, overall, more accurate than Monte-Carlo ANOVA. Additionally, the Monte-Carlo Kruskal-Wallis power study method based on Bernstein fits theoretically has much broader application than ANOVA and a small amount of empirical evidence was provided that this is indeed the case. Of some concern is that both Monte-Carlo methods were found to be considerably more accurate than the analytical method used, which also needs to be confirmed with a larger study. Further large-scale testing is needed in order to delimit the conditions under which accuracy falls.

## Supplementary Information

Supplementary Information.

Supplementary Information.

## Data Availability

Simulation studies: sample values (mydfsample) are stored in datastore files, which also give many other statistics, e.g. in Supplementary file S3B….datastore….RData. Dialysis Study (1): S9A_Dialysis_data.csv, numeric population values also given in S3A_Dialysisrun_Dial_datastore.RData; Diabetes Study (2): the Hispanic Community Health Study from https://sleepdata.org/datasets, NHLBI National Sleep Science Resource^10^; parameters "diabetes2" and "SLPDUR". Marital Study (3): the Sleep Heart Health Study; as for (2), parameters "hdl" and "mstat". All coding and Supplementary files are found at https://github.com/Abiologist/Power.git (or via arxiv: 110.11676).
