# Supplementary material for: Empirical investigations into Kruskal-Wallis power studies utilizing Bernstein fits, simulations and medical study datasets
Source: Sci Rep. 2023 Feb 9;13:2352. doi: 10.1038/s41598-023-29308-2 (PMC9911609; doi:10.1038/s41598-023-29308-2)
Supplement: Supplementary file 1 — Supplementary Information. [file 41598_2023_29308_MOESM1_ESM.docx]

**Supplementary Materials.**

The reader is referred to the on-line Supplementary Materials for complete R coding plus example SLURM coding, "...datastore..." files containing many statistics from method comparisons, and data for the Dialysis study. All supplementary files, figures and all coding are found at https://github.com/Abiologist/Power.git (or via arxiv: 110.11676; to download a file from github: "go to file", click on file, right-click on "raw", "Save Link As"). **Supplementary Files: S1A, S1B, S1C, S1D:** settings files for medical and simulation studies. **S2A, S2B:** sample master and coding. **S2C:** power and power subset masters. **S3:** load coding. **S3A, S3B, S3C, S3D:** datastores. **S4:** power coding files. **S5:** example slurm coding. **S6:** collation coding. **S7, S8:** Monte-Carlo tools - provided as is ! **S9A:** Dialysis data. **S10:** graphical coding. **S11:** parameter definitions for datastores; **S12:** summary medians and Brown-Mood median tests. **Supplementary Figures: S13:S19**.
