# Supplementary material for: Empirical investigations into Kruskal-Wallis power studies utilizing Bernstein fits, simulations and medical study datasets
Source: Sci Rep. 2023 Feb 9;13:2352. doi: 10.1038/s41598-023-29308-2 (PMC9911609; doi:10.1038/s41598-023-29308-2)
Supplement: Supplementary file 2 — Supplementary Information. [file 41598_2023_29308_MOESM2_ESM.pdf]

Supplemental Figures for article:

Empirical Investigations into Kruskal-Wallis Power Studies utilizing Bernstein Fits, Simulations and Medical Study Datasets.

Jeremy S.C. Clark, Piotr Kulig, Konrad Podsiadło, Kamila Rydzewska, Krzysztof Arabski, Monika Białecka, Krzysztof Safranow, Andrzej Ciechanowicz

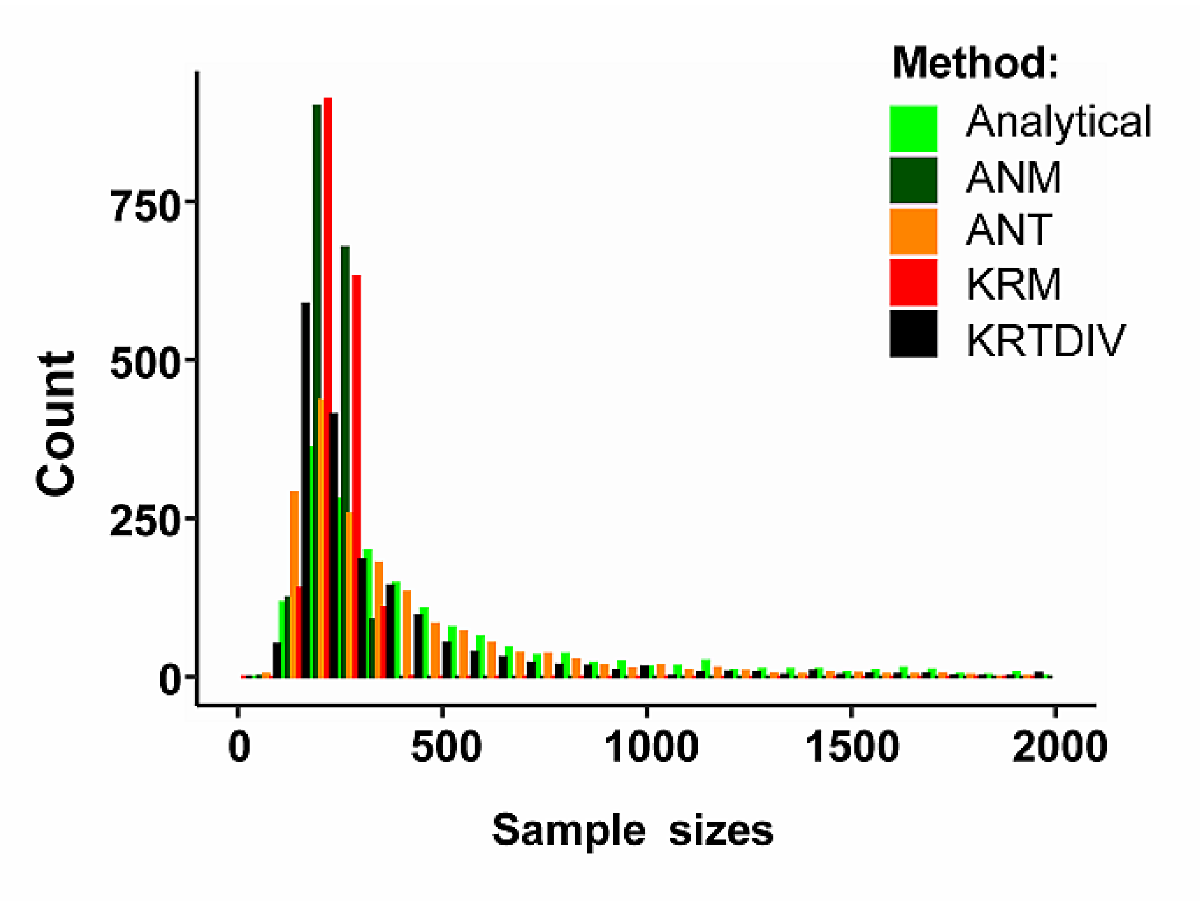

Figure S13. Counts of predicted and measured sample sizes for 80% power in simulation run_three. Power prediction: KRTDIV: Monte-Carlo Kruskal-Wallis power; ANT: Monte-Carlo ANOVA; Analytical: analytical ANOVA. Measured power: KRM: Kruskal-Wallis; ANM: ANOVA. For settings, see Fig 1.

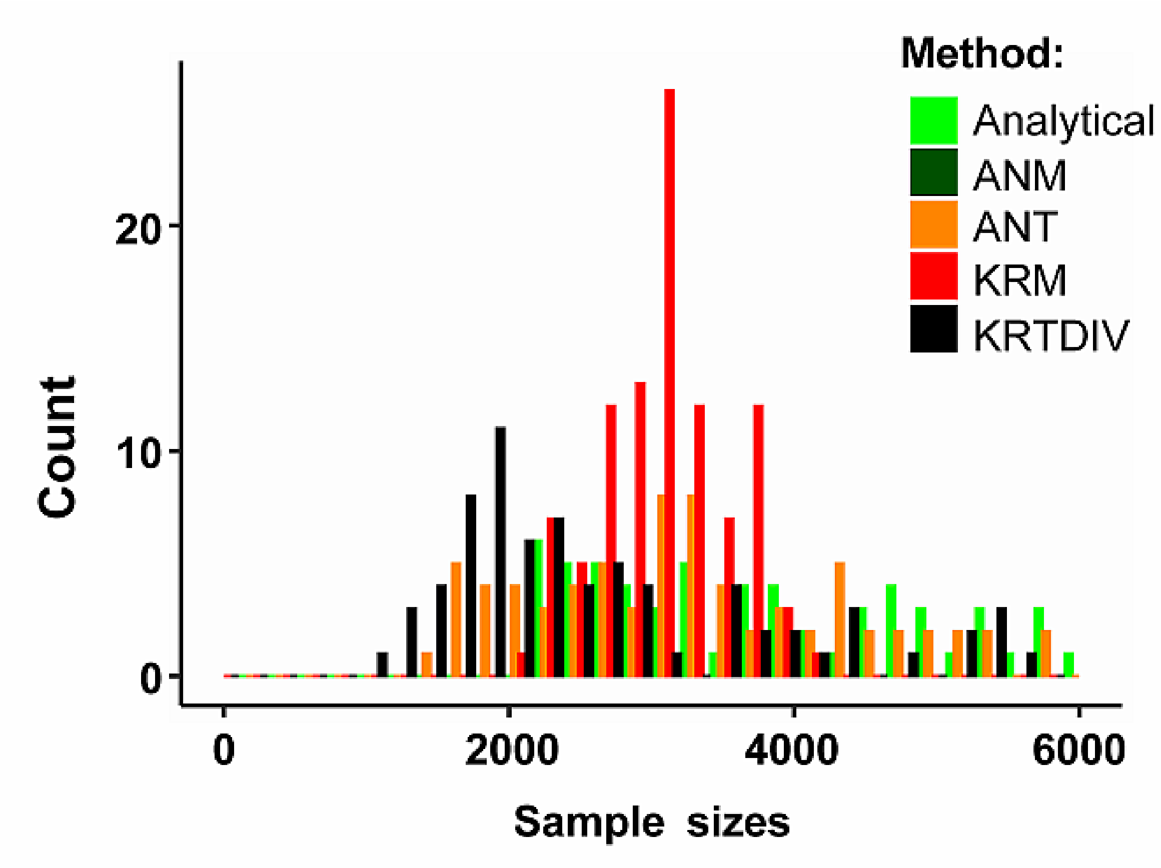

Figure S14. Counts of predicted and measured sample sizes for power in run_DIAB: Diabetes/sleep hours study. For abbreviations see Fig S14.

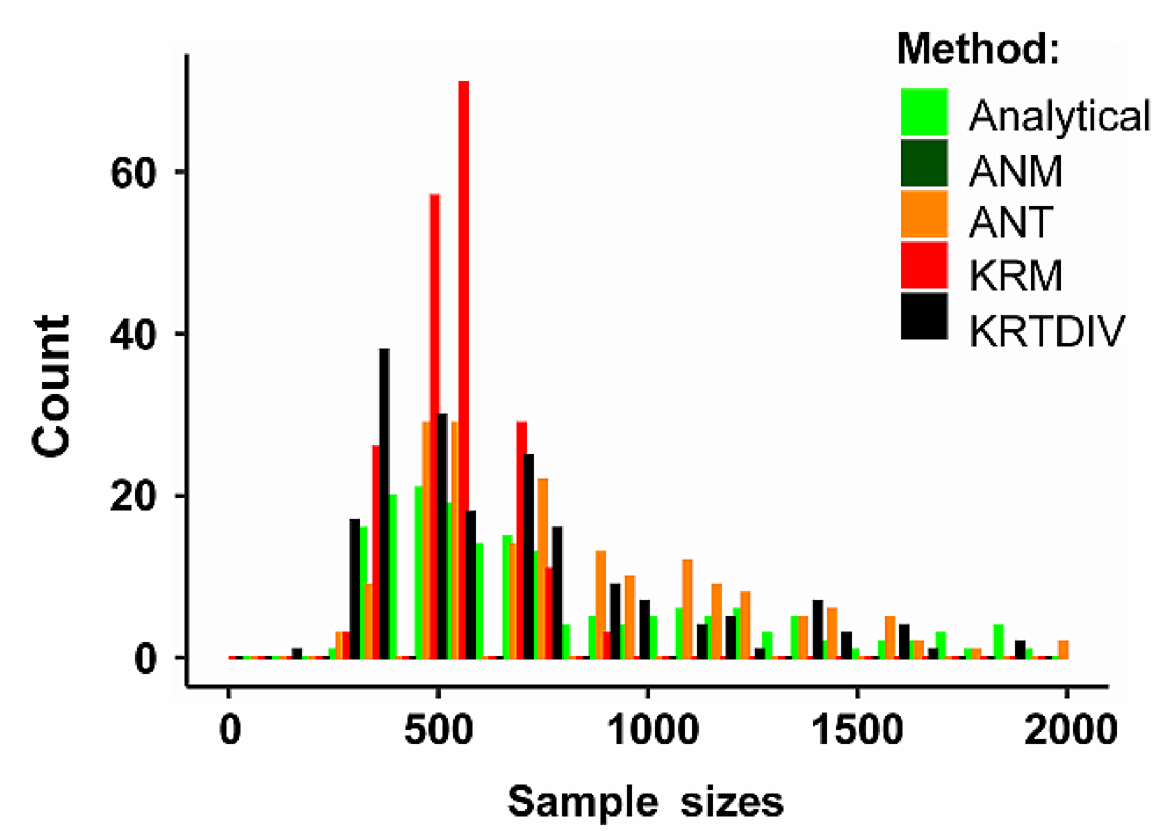

Figure S15. Counts of predicted and measured sample sizes for power in run_Dial: Dialysis/systolic blood pressure study. For abbreviations see Fig S13.

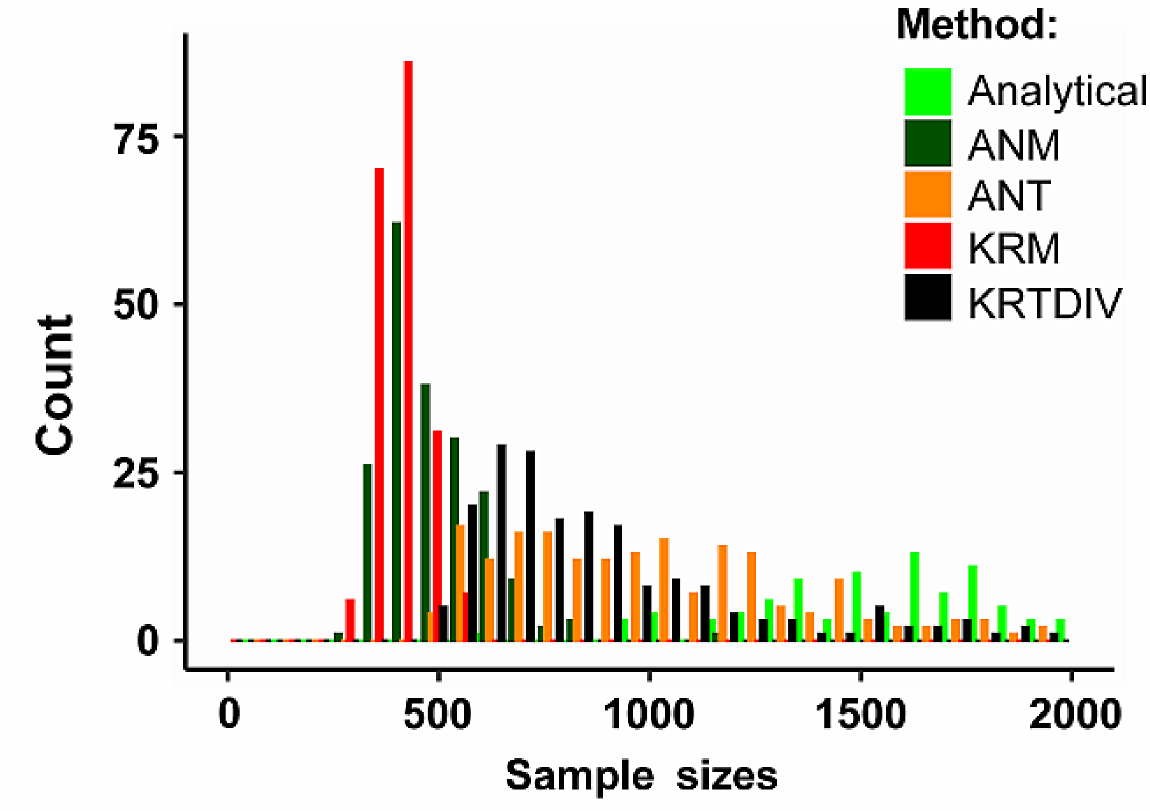

Figure S16. Counts of predicted and measured sample sizes for power in run_Marrelaxed: Marital status/high-density-lipoprotein cholesterol study with relaxed ANOVA conditions. For abbreviations see Fig S13.

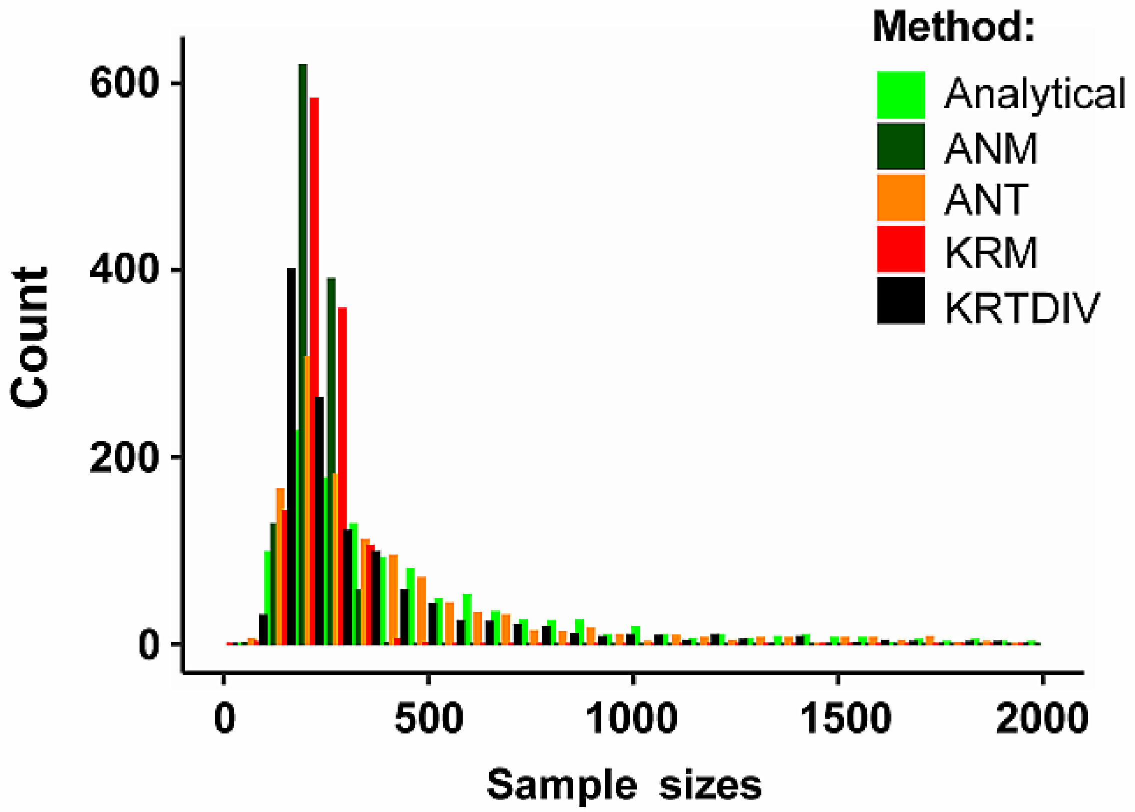

Figure S17. Counts of predicted and measured sample sizes for power in simulation run_one.

For abbreviations see Fig S13; for settings see Fig 2.

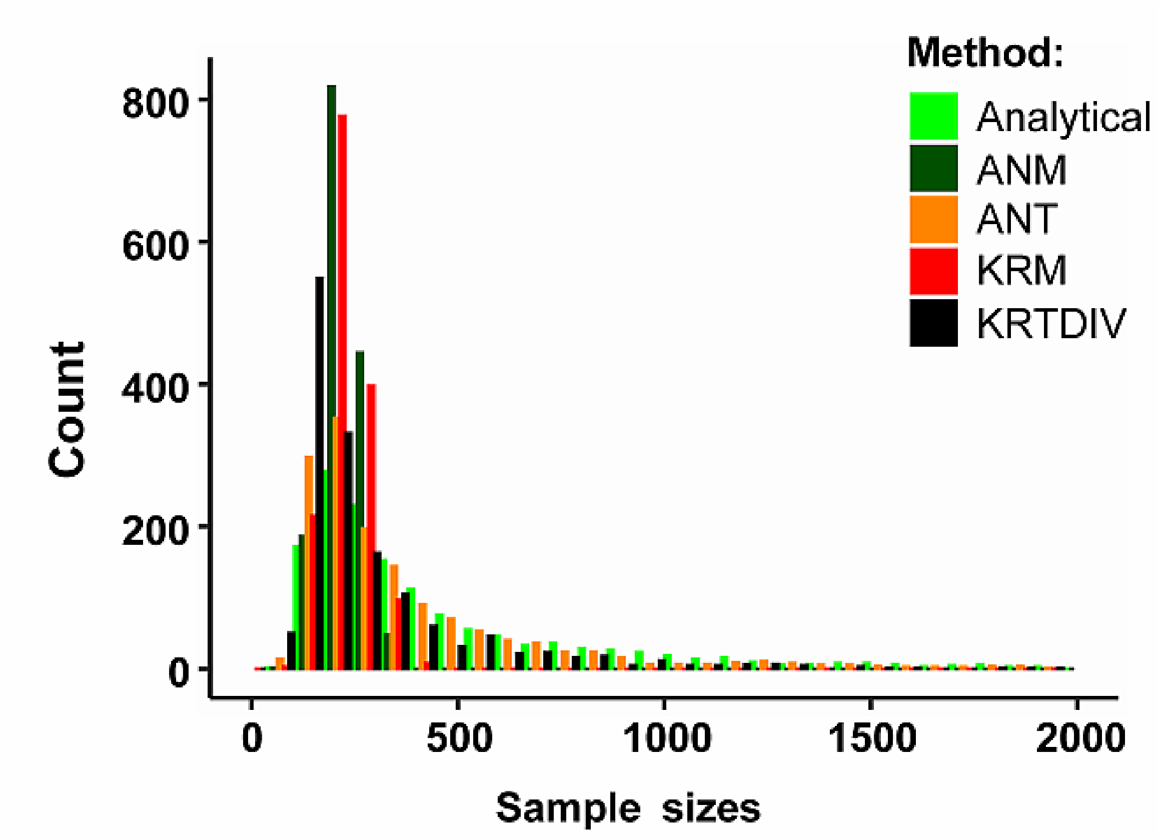

Figure S18. Counts of predicted and measured sample sizes for power in simulation run_one with relaxed ANOVA conditions. For abbreviations see Fig S13; for settings see Fig 3.

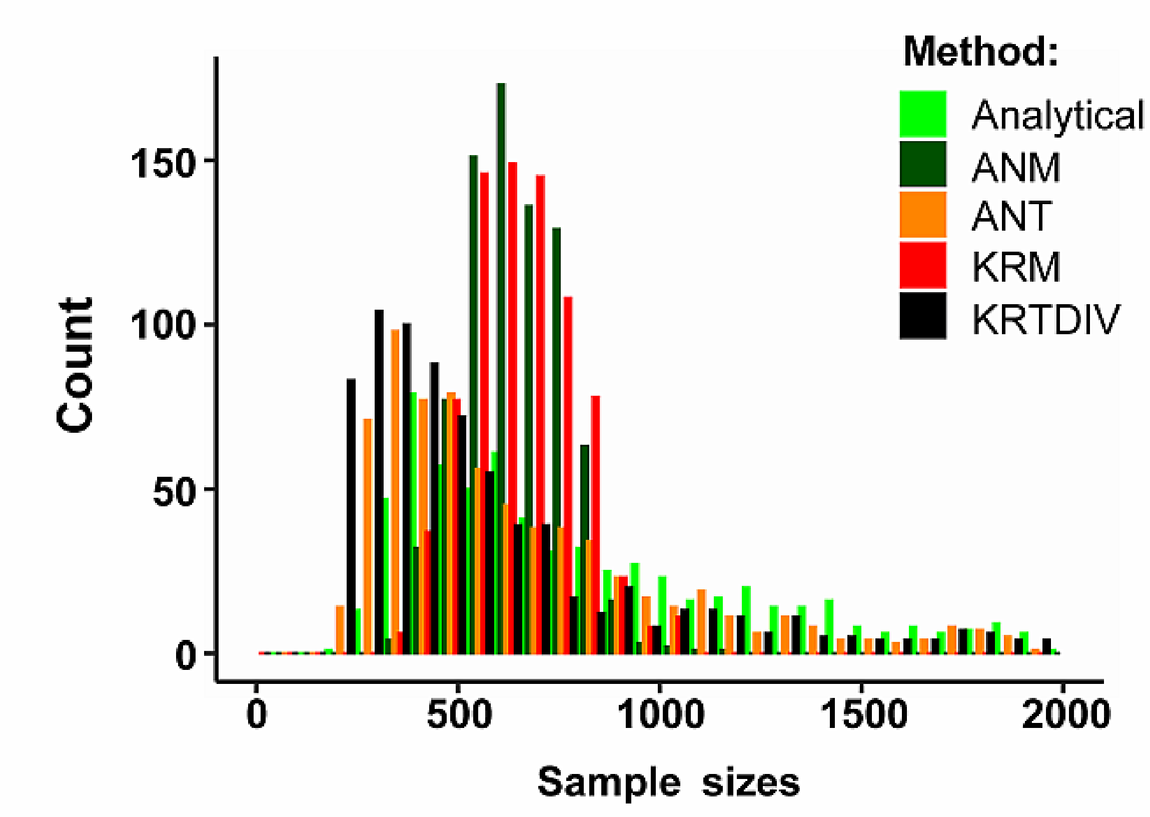

Figure S19. Counts of predicted and measured sample sizes for power in simulation run_two. For abbreviations see Fig S13; for settings see Fig 4.
